# Inhibition of the MID1 protein complex: a novel approach targeting APP protein synthesis

**DOI:** 10.1038/s41420-017-0003-8

**Published:** 2018-01-29

**Authors:** Frank Matthes, Moritz M. Hettich, Judith Schilling, Diana Flores-Dominguez, Nelli Blank, Thomas Wiglenda, Alexander Buntru, Hanna Wolf, Stephanie Weber, Ina Vorberg, Alina Dagane, Gunnar Dittmar, Erich Wanker, Dan Ehninger, Sybille Krauss

**Affiliations:** 1 Deutsches Zentrum für Neurodegenerative Erkrankungen e.V., Bonn, Germany; 2 Max Delbrueck Center for Molecular Medicine (MDC) Berlin-Buch, Berlin, Germany; 3 Luxembourg Institute of Health, Strassen, Luxembourg

## Abstract

Alzheimer’s disease (AD) is characterized by two neuropathological hallmarks: senile plaques, which are composed of amyloid-β (Aβ) peptides, and neurofibrillary tangles, which are composed of hyperphosphorylated tau protein. Aβ peptides are derived from sequential proteolytic cleavage of the amyloid precursor protein (APP). In this study, we identified a so far unknown mode of regulation of APP protein synthesis involving the MID1 protein complex: MID1 binds to and regulates the translation of APP mRNA. The underlying mode of action of MID1 involves the mTOR pathway. Thus, inhibition of the MID1 complex reduces the APP protein level in cultures of primary neurons. Based on this, we used one compound that we discovered previously to interfere with the MID1 complex, metformin, for in vivo experiments. Indeed, long-term treatment with metformin decreased APP protein expression levels and consequently Aβ in an AD mouse model. Importantly, we have initiated the metformin treatment late in life, at a time-point where mice were in an already progressed state of the disease, and could observe an improved behavioral phenotype. These findings together with our previous observation, showing that inhibition of the MID1 complex by metformin also decreases tau phosphorylation, make the MID1 complex a particularly interesting drug target for treating AD.

## Introduction

Alzheimer’s disease (AD), the most common form of dementia in the elderly, is characterized by two neuropathological hallmarks: senile plaques, which are composed of Aβ peptides, and neurofibrillary tangles, which are composed of hyperphosphorylated tau protein. The disease was first described in 1907 by Alois Alzheimer^1^, who observed these two pathological hallmarks in patients’ brains. Aβ peptides are derived from sequential proteolytic cleavage of the amyloid precursor protein (APP). While the non-amyloidogenic pathway involves sequential cleavage of full-length APP by the α-secretases and γ-secretase, the amyloidogenic pathway causing the production of Aβ peptides requires the cleavage of full-length APP by the β-secretase BACE1 and the γ-secretase^2^. Multiple lines of evidence suggest that overproduction of Aβ results in neuronal dysfunction and, finally, in neuronal loss^3^. The second pathological hallmark of AD, neurofibrillary tangles, are mainly composed of hyperphosphorylated tau protein^4,5^. Tau is a microtubule-associated protein that stimulates and stabilizes microtubule assembly. Upon hyperphosphorylation, tau dissociates from microtubules, resulting in microtubule destabilization and neuronal death. The main tau phosphatase is protein phosphatase 2A (PP2A), which is capable of dephosphorylating tau at AD-relevant phospho-sites^6^.

As we have shown previously, the MID1-PP2A protein complex regulates the phosphorylation of tau^7^. MID1 acts as an E3 ubiquitin ligase and promotes the ubiquitin-dependent degradation of PP2A^8^. Therefore, MID1 is a negative regulator of PP2A activity and thus inhibition of the MID1-PP2A complex is a promising approach to activate PP2A, and thereby induce its activity towards its target protein tau. In line with this, we have shown previously that the anti-diabetic drug metformin is capable of dephosphorylating tau at AD-relevant phospho-sites by interfering with the assembly of the MID1-PP2A-complex^7^. Upon metformin treatment, the MID1-dependent degradation of PP2Ac is inhibited, resulting in increased PP2A activity and dephosphorylation of tau at AD specific sites^7^. Besides regulating PP2A activity, MID1 also regulates the activity of the PP2A opposing kinase mTOR^9^. Both enzymes, PP2A and mTOR, play a crucial role in the regulation of translation initiation by the eukaryotic initiation factor (eIF) complex. In detail, in absence of mTOR, a negative regulatory protein complex containing 4E-BP1 in association with eIF4E binds to the 5′ end of the mRNA and inhibits translation. To activate translation mTOR phosphorylates 4E-BP1, thereby releasing its inhibitory action and allowing a heterotrimeric complex containing eIF4E, eIF4A, and eIF4G to assemble at the 5′ end of the mRNA. At the same time, mTOR also phosphorylates and thereby activates p70 S6 kinase (S6K). S6K in turn phosphorylates and thereby activates its downstream targets eIF4B and S6. S6 is part of the small ribosomal subunit (40S). eIF3 is a multi-subunit protein that recruits 40S to the mRNA. Once associated with the mRNA, 40S starts scanning towards the ATG start codon. Upon start codon recognition, the large ribosomal subunit (60S) binds and, together with 40S, forms the translationally competent ribosome. The eIF complex is released from the mRNA and translation starts^10^. PP2A and mTOR control translation by regulating the phosphorylation of 4E-BP1 and S6K. MID1 binds to GC-rich mRNAs and recruits its interacting proteins, including S6K and S6^11,12^. By regulating the activity of both PP2A and mTOR, MID1 controls the translation of mRNAs bound to the MID1 complex^11–14^.

In this study we asked if MID1, besides its regulatory action on phospho-tau, could also affect APP. We show here a so far unknown connection between MID1 and APP: MID1 binds to the APP mRNA and regulates its translation. The underlying mode of action of MID1 involves the mTOR-dependent translation initiation pathway. Furthermore, we used metformin, a compound that we had shown previously to interfere with the MID1 complex and inactivate translation of MID1-target mRNAs^7,14,15^, for a chronic treatment of an AD mouse model at a progressed state of disease. Our data show that metformin treatment decreases the protein levels of APP and consequently Aβ. This together with our previous observation that disassembly of the MID1 protein complex by metformin also decreases tau-phosphorylation^7^, makes the MID1 protein complex a particularly interesting drug target for treating AD.

## Results

### Translation of APP is regulated by MID1

**Fig. 1:**
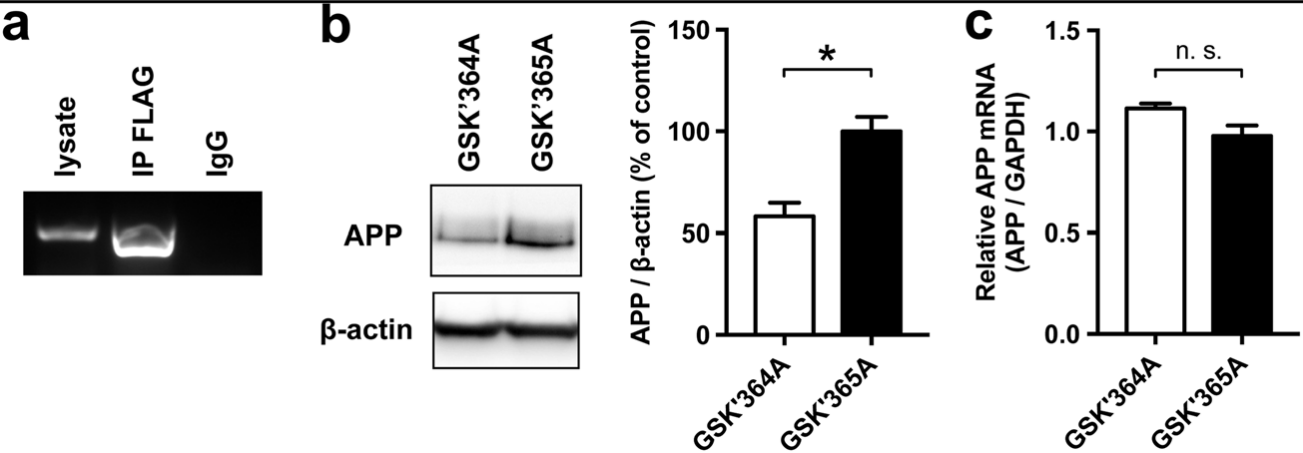
APP is a MID1 target mRNA **a** RNA immunoprecipitation. Primary neurons were transfected with MID1-FLAG. Afterwards MID1-mRNPs were purified by immunoprecipitation (IP FLAG) and MID1-bound mRNAs were analyzed for the presence of APP mRNA using RT-PCR. Unspecific IgG beads (IgG) were used as negative control. 5 µl of the PCR product were analyzed on a 1% agarose gel. **b** Inhibition of the MID1 complex reduces APP protein level. Primary cortical neurons were treated with a peptide that mimics the MID1-α4 binding site and thus outcompetes MID1 (GSK’364A). A mutant peptide was used as negative control (GSK’365A). APP protein levels were analyzed on western blots using APP-specific antibodies. β-actin was detected as loading control. A representative blot of *n* = 3 is shown. Graphs show quantification of western blots, mean values  ± SEM. *p < 0.05. **c** Relative APP mRNA expression was measured in cells treated as in **b** by means of real-time PCR. Columns represent mean values ± SEM. *n* = 3. APP amyloid precursor protein

To investigate if the APP mRNA is regulated by the MID1-PP2A complex, we first tested if MID1 is able to bind to APP mRNA. For this we performed RNA-immunoprecipitations. Primary cortical neurons of wildtype mice were transfected with FLAG-MID1. After UV-crosslinking FLAG-MID1 was purified and MID1-bound mRNAs were isolated from the immunoprecipitates. As negative control an immunoprecipitation using unspecific IgGs was performed. RT-PCRs clearly showed the presence of APP mRNA in the MID1-immunoprecipitates (Fig. 1a).

In previous studies we showed that binding of the MID1-complex to its target mRNAs induces protein translation from the respective mRNA^12,15,16^. Therefore, we asked if the observed binding between APP mRNA and MID1 leads to an induction of APP translation. To test this we performed an experiment in primary cortical neurons with a peptide that mimics the binding sequence between MID1 and the α4-PP2A complex and therefore specifically outcompetes MID1. Depletion of MID1 led to a significant reduction of APP protein as shown on a western blot (Fig. 1b). Of note, the mRNA level of APP did not change significantly (Fig. 1c), which is in line with an inhibition at the protein synthesis level.

### MID1 regulates APP translation by interacting with the mTOR-dependent translation initiation pathway

**Fig. 2:**
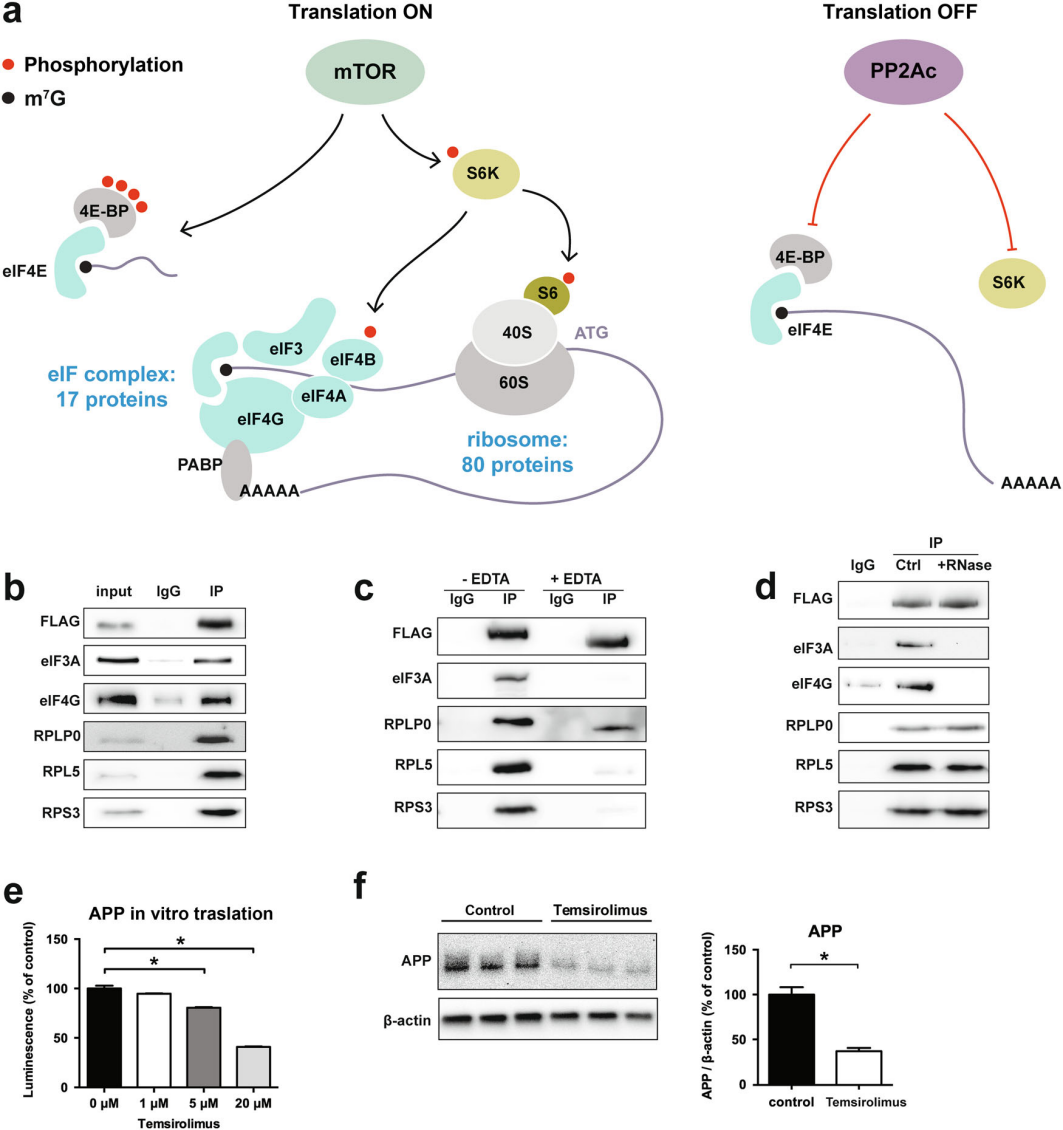
MID1 is connected to the mTOR-dependent translation initiation pathway **a** Identification of the MID1-interactome. MID1-FLAG was expressed in HEK293T cells and MID1-complexes were purified by immunoprecipitation. MID1-binding proteins were identified by mass spectrometry. The mTOR-dependent translation initiation pathway is shown and the number of proteins identified belonging either to the eukaryotic translation initiation factor complex (eIF complex) or the ribosome are indicated. **b** Validation of the mass spectrometry results shown in **a** and Table 1. MID1-FLAG was expressed in HEK293T cells and MID1-complexes were purified by immunoprecipitation (IP FLAG). As negative control, unspecific IgG agarose beads were used (IgG). Immunoprecipitates were analyzed on western blots using specific antibodies to detect MID1-FLAG, eIF3A, eIF4G, RPLP0, RPL5, RPS3. **c** Effect of ribosome disassembly on the composition of the MID1-complex. MID1-FLAG was expressed in HEK293T cells and immunopurified (IP FLAG) in the presence or absence of high concentrations of EDTA. Immunoprecipitates were analyzed on western blots using specific antibodies for MID1-FLAG, eIF3A, RPLP0, RPL5, RPS3. **d** To analyze the MID1-complex composition and its dependency on RNA, MID1-FLAG was expressed in HEK293T cells and immunopurified (IP FLAG) in the presence or absence of RNAse. As negative control, unspecific IgG agarose beads were used (IgG). Immunoprecipitates were analyzed on western blots using specific antibodies for MID1-FLAG, eIF3A, eIF4G, RPLP0, RPL5, RPS3. **e**, **f** mTOR regulates translation of APP. **e** In vitro translation of in vitro transcribed APP-mRNA tagged to luciferase in the presence or absence of the mTOR-inhibitor temsirolimus. The level of translated luciferase reporter was measured in a luciferase assay. Columns represent mean values ± SEM. *n* = 3. *p < 0.01. **f** Primary neurons were treated with the mTOR-inhibitor temsirolimus. Protein extracts were analyzed on western blots, detecting APP and β-actin as loading control. Graph shows quantification of western blots, mean values ± SEM. *n* = 3. *p < 0.01. APP amyloid precursor protein

As we have shown previously, the MID1-complex regulates translation in concert with mTOR. To identify at which exact step of the mTOR-dependent translation initiation MID1 functions, we analyzed proteins that bind to MID1. For this we performed an immunoprecipitation of FLAG-MID1 and analyzed all MID1-bound proteins by mass spectrometry. As expected, we detected several members of the mTOR-translation initiation cascade (Fig. 2a and Table 1), several of which we validated on a western blot of FLAG-MID1-immunoprecipitates using specific antibodies (Fig. 2b). Interestingly, all MID1-bound proteins identified here were proteins of the mTOR-dependent translation pathway that act downstream of mTOR as well as S6K and 4E-BP, suggesting that MID1 regulates the mTOR-dependent translation by acting on either the eIFs or the ribosome. Thus, in a second set of experiments we added EDTA during the co-immunoprecipitation. EDTA dissociates ribosomal particles^17^. While binding of most of the identified proteins to MID1 was abolished by the treatment, RPLP0, a member of the large subunit of the ribosome remained attached to MID1 (Fig. 2c). Since all of the proteins identified by mass spectrometry bind RNA in general, their presence in the MID1-immunoprecipitate may be explained by RNA-mediated indirect binding rather than by direct protein-protein interaction. To test which of the identified proteins bind to MID1 independent of RNA, we performed co-immunoprecipitation experiments in the presence or absence of RNase. Binding of the identified eIF proteins to MID1 was abolished by RNase treatment, while RPLP0, RPL5, and RPS3 remained attached to MID1 (Fig. 2d). These data suggest that MID1 directly binds to the ribosome to stimulate mTOR-dependent translation. In line with a MID1-mTOR dependent translation of APP, application of an mTOR inhibitor reduced APP translation both in an in vitro translation assay (Fig. 2e) as well as in primary neurons (Fig. 2f).

**Table 1:** Statistical analysis of proteins identified mass spectrometry analysis of MID1 immunoprecipitates

Protein name	Gene ID	Log2 ratio	*P*-value
ATP-binding cassette sub-family D member 3	ABCD3	2.75E+01	2.04E-03
ATP-binding cassette sub-family F member 2	ABCF2	2.88E+01	5.96E-04
Apoptotic chromatin condensation inducer in the nucleus	ACIN1	2.72E+01	1.39E-02
Aldehyde dehydrogenase X, mitochondrial	ALDH1B1	2.62E+01	3.61E-04
Mitochondrial 10-formyltetrahydrofolate dehydrogenase	ALDH1L2	2.66E+01	1.97E-02
THO complex subunit 4	ALYREF	2.99E+01	7.55E-04
Serine/threonine-protein phosphatase 6 reg. ankyrin repeat subunit A	ANKRD28	2.63E+01	1.85E-02
Coatomer subunit delta	ARCN1	2.65E+01	2.36E-02
Activating signal cointegrator 1 complex subunit 3	ASCC3	2.59E+01	7.96E-03
ATPase family AAA domain-containing protein 3A	ATAD3A	1.77E+00	3.98E-02
Sodium/potassium-transporting ATPase subunit alpha-1	ATP1A1	2.72E+01	4.39E-04
Ribosome biogenesis protein BMS1 homolog	BMS1	2.90E+01	8.92E-03
Ribosome biogenesis protein BRX1 homolog	BRIX1	2.68E+01	1.41E-02
Caprin-1	CAPRIN1	2.80E+01	5.88E-03
Coiled-coil domain-containing protein 124	CCDC124	2.94E+01	1.91E-04
T-complex protein 1 subunit gamma	CCT3	2.63E+01	4.86E-02
T-complex protein 1 subunit epsilon	CCT5	2.64E+01	1.42E-03
Cell division cycle 5-like protein	CDC5L	3.04E+01	3.51E-03
Centrosomal protein of 170 kDa	CEP170	2.71E+01	2.53E-03
Chromatin target of PRMT1 protein	CHTOP	2.81E+01	3.45E-02
CLIP-associating protein 2	CLASP2	2.74E+01	4.83E-03
Methylosome subunit pICln	CLNS1A	2.97E+01	2.16E-03
Coatomer subunit gamma-2	COPG2	2.59E+01	2.98E-04
Coronin-1C	CORO1C	3.03E+01	2.00E-02
Cleavage and polyadenylation specificity factor subunit 6	CPSF6	2.93E+01	4.13E-03
Cleavage and polyadenylation specificity factor subunit 7	CPSF7	2.73E+01	2.20E-03
Probable ATP-dependent RNA helicase DDX17	DDX17	3.22E+01	1.28E-02
Probable ATP-dependent RNA helicase DDX20	DDX20	2.73E+01	2.81E-02
Nucleolar RNA helicase 2	DDX21	3.01E+01	8.42E-03
Probable ATP-dependent RNA helicase DDX23	DDX23	2.93E+01	4.58E-03
ATP-dependent RNA helicase DDX3X	DDX3X	2.66E+01	5.42E-04
Probable ATP-dependent RNA helicase DDX41	DDX41	2.73E+01	4.88E-03
Probable ATP-dependent RNA helicase DDX5	DDX5	2.97E+01	1.38E-02
ATP-dependent RNA helicase DDX50	DDX50	2.65E+01	5.75E-03
Putative pre-mRNA-splicing factor ATP-dependent RNA helicase DHX15	DHX15	2.76E+01	6.67E-03
Putative ATP-dependent RNA helicase DHX30	DHX30	2.99E+01	1.03E-02
ATP-dependent RNA helicase A	DHX9	7.63E+00	4.50E-03
Elongation factor 2	EEF2	4.68E+00	8.14E-03
116 kDa U5 small nuclear ribonucleoprotein component	EFTUD2	3.04E+01	6.54E-03
Eukaryotic translation initiation factor 3 subunit A	EIF3A	3.34E+01	2.89E-04
Eukaryotic translation initiation factor 3 subunit B	EIF3B	3.19E+01	6.22E-03
Eukaryotic translation initiation factor 3 subunit C	EIF3C	3.23E+01	1.43E-03
Eukaryotic translation initiation factor 3 subunit D	EIF3D	3.01E+01	1.72E-03
Eukaryotic translation initiation factor 3 subunit E	EIF3E	3.15E+01	2.60E-03
Eukaryotic translation initiation factor 3 subunit F	EIF3F	3.08E+01	9.00E-03
Eukaryotic translation initiation factor 3 subunit G	EIF3G	2.99E+01	1.64E-04
Eukaryotic translation initiation factor 3 subunit I	EIF3I	3.07E+01	4.95E-03
Eukaryotic translation initiation factor 3 subunit J	EIF3J	2.75E+01	2.97E-04
Eukaryotic translation initiation factor 3 subunit K	EIF3K	2.75E+01	4.35E-02
Eukaryotic translation initiation factor 3 subunit L	EIF3L	3.20E+01	1.62E-03
Eukaryotic translation initiation factor 3 subunit M	EIF3M	3.06E+01	2.52E-02
Eukaryotic translation initiation factor 3 subunit H	EIF3S3	2.98E+01	4.34E-03
Eukaryotic initiation factor 4A-I	EIF4A1	2.82E+01	1.28E-02
Eukaryotic initiation factor 4A-III	EIF4A3	2.66E+01	2.37E-02
Eukaryotic translation initiation factor 4B	EIF4B	3.07E+01	1.05E-02
Eukaryotic translation initiation factor 6	EIF6	2.71E+01	1.74E-02
Emerin	EMD	2.73E+01	1.99E-02
Erlin-2	ERLIN2	2.81E+01	7.54E-03
Exosome component 10	EXOSC10	2.76E+01	3.45E-03
Exosome complex component RRP45	EXOSC9	2.59E+01	2.53E-02
Constitutive coactivator of PPAR-gamma-like protein 1	FAM120A	2.74E+01	1.36E-02
Phenylalanine--tRNA ligase alpha subunit	FARSA	2.78E+01	7.54E-03
Phenylalanine--tRNA ligase beta subunit	FARSB	2.78E+01	1.17E-02
40S ribosomal protein S30	FAU	2.85E+01	2.31E-03
Protein furry homolog-like	FRYL	3.02E+01	2.54E-02
Gem-associated protein 4	GEMIN4	2.71E+01	2.53E-03
Guanine nucleotide-binding protein subunit beta-2-like 1	GNB2L1	3.25E+01	3.47E-04
Nucleolar GTP-binding protein 2	GNL2	2.75E+01	7.11E-03
Guanine nucleotide-binding protein-like 3	GNL3	2.69E+01	4.03E-04
Golgin subfamily A member 3	GOLGA3	2.93E+01	3.96E-03
General transcription factor 3C polypeptide 2	GTF3C2	2.75E+01	3.97E-03
General transcription factor 3C polypeptide 3	GTF3C3	2.57E+01	3.56E-02
General transcription factor 3C polypeptide 4	GTF3C4	2.70E+01	2.46E-03
Nucleolar GTP-binding protein 1	GTPBP4	2.87E+01	1.26E-02
Histone H2B	HIST1H2BN	2.99E+01	1.62E-02
Heterogeneous nuclear ribonucleoproteins C1/C2	HNRNPC	3.08E+01	3.22E-04
Heterogeneous nuclear ribonucleoprotein D0	HNRNPD	2.60E+01	2.60E-02
Heterogeneous nuclear ribonucleoprotein F	HNRNPF	2.74E+01	4.67E-03
Heterogeneous nuclear ribonucleoprotein K	HNRNPK	2.96E+01	9.43E-04
Heterogeneous nuclear ribonucleoprotein M	HNRNPM	6.56E+00	1.98E-02
Heterogeneous nuclear ribonucleoprotein R	HNRNPR	2.99E+01	4.29E-03
Heterogeneous nuclear ribonucleoprotein U	HNRNPU	3.23E+01	2.34E-03
Isoleucine--tRNA ligase, cytoplasmic	IARS	2.72E+01	3.12E-03
Insulin-like growth factor 2 mRNA-binding protein 1	IGF2BP1	3.18E+01	1.09E-03
Insulin-like growth factor 2 mRNA-binding protein 3	IGF2BP3	2.81E+01	1.22E-02
Interleukin enhancer-binding factor 2	ILF2	3.10E+01	1.08E-02
Interleukin enhancer-binding factor 3	ILF3	3.27E+01	2.99E-03
Importin-8	IPO8	2.74E+01	1.30E-02
Insulin receptor substrate 4	IRS4	1.33E+00	3.38E-03
Influenza virus NS1A-binding protein	IVNS1ABP	3.33E+01	3.05E-03
Tyrosine-protein kinase JAK1	JAK1	2.88E+01	4.64E-03
BTB/POZ domain-containing protein KCTD17	KCTD17	2.96E+01	1.23E-02
BTB/POZ domain-containing protein KCTD5	KCTD5	2.96E+01	3.67E-04
Kinesin-like protein KIF11	KIF11	1.48E+00	1.25E-03
La-related protein 1	LARP1	3.17E+01	9.37E-04
La-related protein 4	LARP4	2.86E+01	7.22E-03
La-related protein 4B	LARP4B	2.65E+01	8.21E-03
LIM domain and actin-binding protein 1	LIMA1	2.98E+01	7.34E-03
Leucine-rich PPR motif-containing protein, mitochondrial	LRPPRC	2.63E+01	8.82E-03
Putative RNA-binding protein Luc7-like 2	LUC7L2	3.00E+01	3.39E-03
Luc7-like protein 3	LUC7L3	2.81E+01	5.20E-03
Microtubule-associated protein 1B	MAP1B	3.01E+01	3.76E-03
Serine/threonine-protein kinase MARK2	MARK2	2.60E+01	3.76E-03
Methionine--tRNA ligase, cytoplasmic	MARS	2.62E+01	1.62E-03
Matrin-3	MATR3	2.87E+01	1.32E-03
DNA replication licensing factor MCM7	MCM7	2.83E+01	8.19E-03
E3 ubiquitin-protein ligase Midline-1	MID1	3.76E+01	3.26E-04
Putative helicase MOV-10	MOV10	2.77E+01	2.49E-02
28S ribosomal protein S17, mitochondrial	MRPS17	2.91E+01	6.96E-03
28S ribosomal protein S22, mitochondrial	MRPS22	2.74E+01	4.69E-03
28S ribosomal protein S25, mitochondrial	MRPS25	2.71E+01	1.14E-02
28S ribosomal protein S27, mitochondrial	MRPS27	2.64E+01	3.48E-02
Protein LYRIC	MTDH	2.72E+01	1.03E-02
Myb-binding protein 1A	MYBBP1A	2.90E+01	1.44E-04
Myosin-10	MYH10	1.13E+00	2.28E-02
Myosin-9	MYH9	2.92E+01	4.78E-03
Unconventional myosin-Ib	MYO1B	2.71E+01	2.88E-02
Nicotinamide phosphoribosyltransferase	NAMPT	2.75E+01	8.55E-03
Nucleosome assembly protein 1-like 1	NAP1L1	2.57E+01	7.18E-03
Nuclear cap-binding protein subunit 1	NCBP1	2.81E+01	4.89E-04
Nucleolin	NCL	2.86E+01	6.74E-03
Nucleolar complex protein 4 homolog	NOC4L	2.80E+01	8.27E-03
Probable 28S rRNA (cytosine(4447)-C(5))-methyltransferase	NOP2	2.62E+01	5.08E-03
Cleavage and polyadenylation specificity factor subunit 5	NUDT21	2.96E+01	6.21E-04
OTU domain-containing protein 4	OTUD4	2.74E+01	1.45E-03
Prolyl 4-hydroxylase subunit alpha-1	P4HA1	2.79E+01	4.44E-03
Proliferation-associated protein 2G4	PA2G4	3.08E+01	7.55E-03
Polyadenylate-binding protein 1	PABPC1	3.23E+01	2.17E-03
Polyadenylate-binding protein 4	PABPC4	3.22E+01	2.78E-03
Programmed cell death protein 4	PDCD4	2.94E+01	8.37E-03
Proline-, glutamic acid- and leucine-rich protein 1	PELP1	2.66E+01	9.09E-03
Serine/threonine-protein phosphatase PGAM5, mitochondrial	PGAM5	2.86E+01	3.52E-03
Protein arginine N-methyltransferase 5	PRMT5	3.54E+01	6.16E-04
Pre-mRNA-processing factor 19	PRPF19	3.02E+01	3.67E-03
U4/U6 small nuclear ribonucleoprotein Prp3	PRPF3	2.70E+01	1.69E-02
U4/U6 small nuclear ribonucleoprotein Prp31	PRPF31	2.97E+01	8.19E-04
U4/U6 small nuclear ribonucleoprotein Prp4	PRPF4	2.64E+01	3.83E-03
Pre-mRNA-processing factor 6	PRPF6	2.98E+01	6.12E-03
Pre-mRNA-processing-splicing factor 8	PRPF8	3.11E+01	3.06E-03
Ribose-phosphate pyrophosphokinase 1	PRPS1	5.58E+00	3.60E-03
Ribose-phosphate pyrophosphokinase 2	PRPS2	2.93E+01	1.56E-02
Phosphoribosyl pyrophosphate synthase-associated protein 1	PRPSAP1	3.05E+01	8.50E-03
Phosphoribosyl pyrophosphate synthase-associated protein 2	PRPSAP2	3.25E+01	2.15E-04
Protein PRRC2A	PRRC2A	2.85E+01	8.43E-03
Protein PRRC2C	PRRC2C	2.97E+01	2.05E-02
26S protease regulatory subunit 4	PSMC1	2.97E+01	5.78E-03
26S protease regulatory subunit 7	PSMC2	3.07E+01	8.06E-04
26S protease regulatory subunit 6A	PSMC3	2.89E+01	5.50E-03
26S protease regulatory subunit 6B	PSMC4	1.19E+00	1.54E-02
26S protease regulatory subunit 8	PSMC5	4.55E+00	3.71E-04
26S protease regulatory subunit 10B	PSMC6	2.84E+01	1.52E-03
26S proteasome non-ATPase regulatory subunit 1	PSMD1	2.97E+01	2.12E-03
26S proteasome non-ATPase regulatory subunit 10	PSMD10	2.85E+01	2.02E-02
26S proteasome non-ATPase regulatory subunit 11	PSMD11	3.02E+01	1.94E-02
26S proteasome non-ATPase regulatory subunit 12	PSMD12	2.90E+01	4.18E-03
26S proteasome non-ATPase regulatory subunit 13	PSMD13	2.95E+01	3.11E-03
26S proteasome non-ATPase regulatory subunit 14	PSMD14	2.80E+01	1.85E-03
26S proteasome non-ATPase regulatory subunit 2	PSMD2	2.15E+00	4.38E-03
26S proteasome non-ATPase regulatory subunit 3	PSMD3	3.04E+01	1.48E-04
26S proteasome non-ATPase regulatory subunit 4	PSMD4	2.79E+01	1.70E-04
26S proteasome non-ATPase regulatory subunit 6	PSMD6	2.96E+01	9.65E-03
26S proteasome non-ATPase regulatory subunit 7	PSMD7	3.75E+00	1.13E-02
26S proteasome non-ATPase regulatory subunit 8	PSMD8	2.75E+01	1.56E-03
Poly(U)-binding-splicing factor PUF60	PUF60	2.75E+01	9.95E-04
Pyrroline-5-carboxylate reductase	PYCR1	2.55E+01	3.49E-03
RNA-binding protein 10	RBM10	3.30E+01	4.88E-04
RNA-binding protein 14	RBM14	2.94E+01	6.57E-03
RNA-binding protein 25	RBM25	2.75E+01	7.71E-03
RNA-binding protein 26	RBM26	2.63E+01	3.79E-02
RNA-binding protein 27	RBM27	2.70E+01	1.20E-02
RNA-binding protein 28	RBM28	2.63E+01	5.02E-03
RNA-binding motif protein, X chromosome	RBMX	3.01E+01	8.90E-03
RNA 3-terminal phosphate cyclase-like protein	RCL1	2.65E+01	1.64E-03
Reticulocalbin-2	RCN2	2.73E+01	2.67E-03
Replication factor C subunit 3	RFC3	2.62E+01	2.34E-02
Telomere-associated protein RIF1	RIF1	3.04E+01	9.16E-03
Serine/threonine-protein kinase RIO1	RIOK1	3.02E+01	3.05E-03
RING finger protein 219	RNF219	2.94E+01	2.50E-03
RNA-binding protein 39	RNPC2	2.86E+01	8.92E-03
60S ribosomal protein L10	RPL10	3.26E+01	7.84E-03
60S ribosomal protein L10a	RPL10A	3.22E+01	2.24E-02
60S ribosomal protein L11	RPL11	3.19E+01	1.04E-03
60S ribosomal protein L12	RPL12	3.18E+01	6.43E-03
60S ribosomal protein L13	RPL13	3.30E+01	4.30E-04
60S ribosomal protein L13a	RPL13A	3.17E+01	2.25E-03
60S ribosomal protein L14	RPL14	3.07E+01	7.88E-04
60S ribosomal protein L15	RPL15	5.67E+00	8.90E-03
60S ribosomal protein L17	RPL17	3.16E+01	4.69E-04
60S ribosomal protein L18	RPL18	3.28E+01	1.68E-03
60S ribosomal protein L18a	RPL18A	3.24E+01	2.10E-03
Ribosomal protein L19	RPL19	3.26E+01	4.36E-03
60S ribosomal protein L21	RPL21	3.11E+01	1.99E-03
60S ribosomal protein L22	RPL22	3.05E+01	1.47E-03
60S ribosomal protein L22-like 1	RPL22L1	2.70E+01	6.60E-03
60S ribosomal protein L23	RPL23	3.09E+01	4.70E-03
60S ribosomal protein L23a	RPL23A	3.24E+01	1.26E-03
60S ribosomal protein L24	RPL24	3.03E+01	1.24E-03
60S ribosomal protein L26	RPL26	3.24E+01	1.20E-03
60S ribosomal protein L27	RPL27	3.18E+01	3.06E-03
60S ribosomal protein L27a	RPL27A	3.06E+01	5.33E-04
60S ribosomal protein L28	RPL28	3.22E+01	6.23E-04
60S ribosomal protein L29	RPL29	3.19E+01	3.76E-04
60S ribosomal protein L3	RPL3	7.15E+00	6.72E-03
60S ribosomal protein L30	RPL30	3.04E+01	2.06E-03
60S ribosomal protein L31	RPL31	3.15E+01	3.12E-04
60S ribosomal protein L32	RPL32	3.18E+01	3.51E-04
60S ribosomal protein L34	RPL34	2.90E+01	4.73E-03
60S ribosomal protein L35	RPL35	3.13E+01	1.43E-02
60S ribosomal protein L35a	RPL35A	3.14E+01	2.01E-02
60S ribosomal protein L36	RPL36	3.02E+01	1.49E-02
60S ribosomal protein L36a	RPL36A	2.97E+01	5.51E-04
60S ribosomal protein L37a	RPL37A	3.07E+01	1.05E-04
60S ribosomal protein L38	RPL38	2.90E+01	3.49E-02
60S ribosomal protein L4	RPL4	3.36E+01	1.82E-03
60S ribosomal protein L5	RPL5	3.26E+01	6.62E-03
60S ribosomal protein L6	RPL6	6.39E+00	9.35E-03
60S ribosomal protein L7	RPL7	3.33E+01	2.21E-03
60S ribosomal protein L7a	RPL7A	8.22E+00	1.74E-03
60S ribosomal protein L8	RPL8	3.33E+01	9.73E-04
60S ribosomal protein L9	RPL9	3.06E+01	1.10E-04
60S acidic ribosomal protein P0	RPLP0	3.23E+01	2.18E-03
60S acidic ribosomal protein P2	RPLP2	2.87E+01	4.32E-03
40S ribosomal protein S10	RPS10	3.21E+01	1.86E-03
40S ribosomal protein S11	RPS11	3.24E+01	1.88E-03
40S ribosomal protein S12	RPS12	3.14E+01	4.05E-04
40S ribosomal protein S13	RPS13	3.22E+01	1.60E-03
40S ribosomal protein S14	RPS14	3.15E+01	4.92E-04
40S ribosomal protein S15	RPS15	3.15E+01	3.33E-02
40S ribosomal protein S15a	RPS15A	3.19E+01	1.70E-03
40S ribosomal protein S16	RPS16	3.24E+01	1.08E-03
40S ribosomal protein S17	RPS17	3.19E+01	1.78E-03
40S ribosomal protein S18	RPS18	8.03E+00	7.90E-09
40S ribosomal protein S19	RPS19	3.22E+01	1.65E-03
40S ribosomal protein S2	RPS2	3.28E+01	1.71E-03
40S ribosomal protein S20	RPS20	3.21E+01	4.89E-04
40S ribosomal protein S21	RPS21	2.88E+01	2.47E-03
40S ribosomal protein S23	RPS23	3.25E+01	1.31E-03
40S ribosomal protein S24	RPS24	3.00E+01	1.10E-04
40S ribosomal protein S25	RPS25	3.14E+01	5.19E-03
40S ribosomal protein S26	RPS26	3.08E+01	2.54E-02
40S ribosomal protein S27	RPS27	3.00E+01	3.65E-03
40S ribosomal protein S3	RPS3	3.28E+01	3.26E-03
40S ribosomal protein S3a	RPS3A	3.30E+01	4.75E-04
40S ribosomal protein S4, X isoform	RPS4X	6.65E+00	2.28E-03
40S ribosomal protein S6	RPS6	3.18E+01	2.28E-03
40S ribosomal protein S7	RPS7	3.28E+01	1.99E-02
40S ribosomal protein S8	RPS8	3.24E+01	3.63E-03
40S ribosomal protein S9	RPS9	3.31E+01	3.94E-03
40S ribosomal protein SA	RPSA	3.32E+01	1.29E-03
Ribosome-binding protein 1	RRBP1	2.84E+01	3.20E-02
RRP12-like protein	RRP12	2.58E+01	1.13E-02
Ribosomal L1 domain-containing protein 1	RSL1D1	2.69E+01	1.56E-02
U4/U6.U5 tri-snRNP-associated protein 1	SART1	2.91E+01	1.18E-04
Splicing factor, arginine/serine-rich 15	SCAF4	2.74E+01	1.68E-02
Protein SDA1 homolog	SDAD1	2.64E+01	1.69E-02
Plasminogen activator inhibitor 1 RNA-binding protein	SERBP1	3.28E+01	2.19E-02
Splicing factor 3B subunit 1	SF3B1	2.81E+01	8.87E-03
Splicing factor 3B subunit 3	SF3B3	2.72E+01	1.14E-02
Superkiller viralicidic activity 2-like 2	SKIV2L2	2.75E+01	8.05E-03
U5 small nuclear ribonucleoprotein 200 kDa helicase	SNRNP200	3.06E+01	5.98E-03
U5 small nuclear ribonucleoprotein 40 kDa protein	SNRNP40	2.72E+01	1.41E-02
Small nuclear ribonucleoprotein Sm D1	SNRPD1	3.02E+01	1.48E-03
Small nuclear ribonucleoprotein Sm D2	SNRPD2	2.92E+01	2.94E-04
Small nuclear ribonucleoprotein Sm D3	SNRPD3	2.95E+01	1.93E-02
Small nuclear ribonucleoprotein-associated proteins B and B	SNRPN	3.06E+01	4.62E-04
Spectrin alpha chain, non-erythrocytic 1	SPTAN1	3.41E+01	9.63E-04
Spectrin beta chain, non-erythrocytic 1	SPTBN1	3.41E+01	9.41E-05
SRSF protein kinase 1	SRPK1	2.94E+01	5.81E-03
SRSF protein kinase 2	SRPK2	2.60E+01	2.88E-02
Serine/arginine repetitive matrix protein 1	SRRM1	2.85E+01	1.46E-02
Serrate RNA effector molecule homolog	SRRT	2.57E+01	2.04E-03
Serine/arginine-rich splicing factor 1	SRSF1	2.74E+01	1.86E-04
Serine/arginine-rich splicing factor 2	SRSF2	2.69E+01	4.59E-02
Serine/arginine-rich splicing factor 3	SRSF3	2.87E+01	1.42E-03
Double-stranded RNA-binding protein Staufen homolog 1	STAU1	2.88E+01	1.18E-02
Serine/threonine-protein kinase 38	STK38	2.78E+01	2.59E-02
SUN domain-containing protein 2	SUN2	2.97E+01	6.35E-04
Heterogeneous nuclear ribonucleoprotein Q	SYNCRIP	2.82E+01	1.08E-02
Very-long-chain enoyl-CoA reductase	TECR	2.70E+01	4.59E-03
Testis-expressed sequence 10 protein	TEX10	2.64E+01	1.64E-03
THO complex subunit 2	THOC2	2.53E+01	1.37E-02
Tight junction protein ZO-2	TJP2	2.65E+01	6.69E-03
Transmembrane protein 33	TMEM33	2.74E+01	1.11E-02
Tropomodulin-3	TMOD3	2.63E+01	4.62E-03
TRMT1-like protein	TRMT1L	2.82E+01	9.34E-03
Tubulin beta-3 chain	TUBB3	2.57E+01	5.04E-04
Tubulin beta-4A chain	TUBB4A	2.61E+01	2.90E-02
Splicing factor U2AF 35 kDa subunit	U2AF1	2.90E+01	7.02E-03
Splicing factor U2AF 65 kDa subunit	U2AF2	2.98E+01	4.30E-03
U2 snRNP-associated SURP motif-containing protein	U2SURP	2.78E+01	1.78E-05
E3 ubiquitin-protein ligase UBR5	UBR5	2.78E+01	2.75E-02
U4/U6.U5 tri-snRNP-associated protein 2	USP39	2.88E+01	2.14E-02
Transitional endoplasmic reticulum ATPase	VCP	2.80E+01	7.61E-05
Vimentin	VIM	1.35E+00	2.08E-02
Methylosome protein 50	WDR77	3.30E+01	2.27E-02
Exportin-T	XPOT	2.64E+01	7.27E-03
Nuclease-sensitive element-binding protein 1	YBX1	3.07E+01	3.00E-03
YTH domain-containing protein 1	YTHDC1	2.78E+01	8.82E-03
YTH domain-containing family protein 2	YTHDF2	1.60E+00	3.42E-02
Zinc finger CCCH domain-containing protein 18	ZC3H18	2.79E+01	1.14E-02
Zinc finger CCCH-type antiviral protein 1	ZC3HAV1	2.79E+01	1.27E-02
Zinc finger protein 622	ZNF622	2.75E+01	1.14E-02

Log2 ratio and *p*-values were calculated using measured protein intensities, i.e. eXtracted Ion Current (XIC) of all isotopic clusters associated with the identified amino acid sequence. Log2 ratio was calculated from the intensity sum of samples/ controls. *p*-values are the result of a two-sided t-test, samples vs. control. In cases where intensities had been measured in 2 (out of 3) replicates, the third intensity value was added through imputation. If no intensity could be measured in all 3 replicates, the intensities were set from 0 to 1 in order to still be able to calculate a ratio (same applies to cases where only 1 intensity could be measured)

### Metformin reduces APP protein and APP cleavage products

**Fig. 3:**
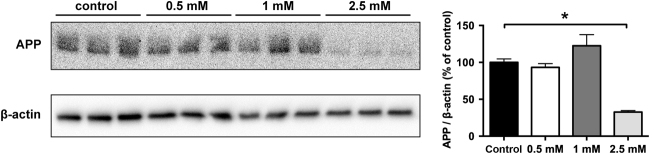
Disruption of the MID1-complex by metformin reduces APP protein level Primary neurons were treated with different doses of metformin. Protein extracts were analyzed on western blots detecting APP and β-actin as loading control. Graph shows quantification of western blots, mean values ± SEM. *n* = 3 *p < 0.01. APP amyloid precursor protein

To target MID1 we decided to use metformin, a compound that we had shown previously to interfere with the MID1 complex and inactivate translation of MID1-target mRNAs^7,14,15^. In line with what we have observed for other MID1-target mRNAs, metformin treatment of primary cortical neurons also led to a reduction of APP protein in a dose-dependent manner (Fig. 3a).

**Fig. 4:**
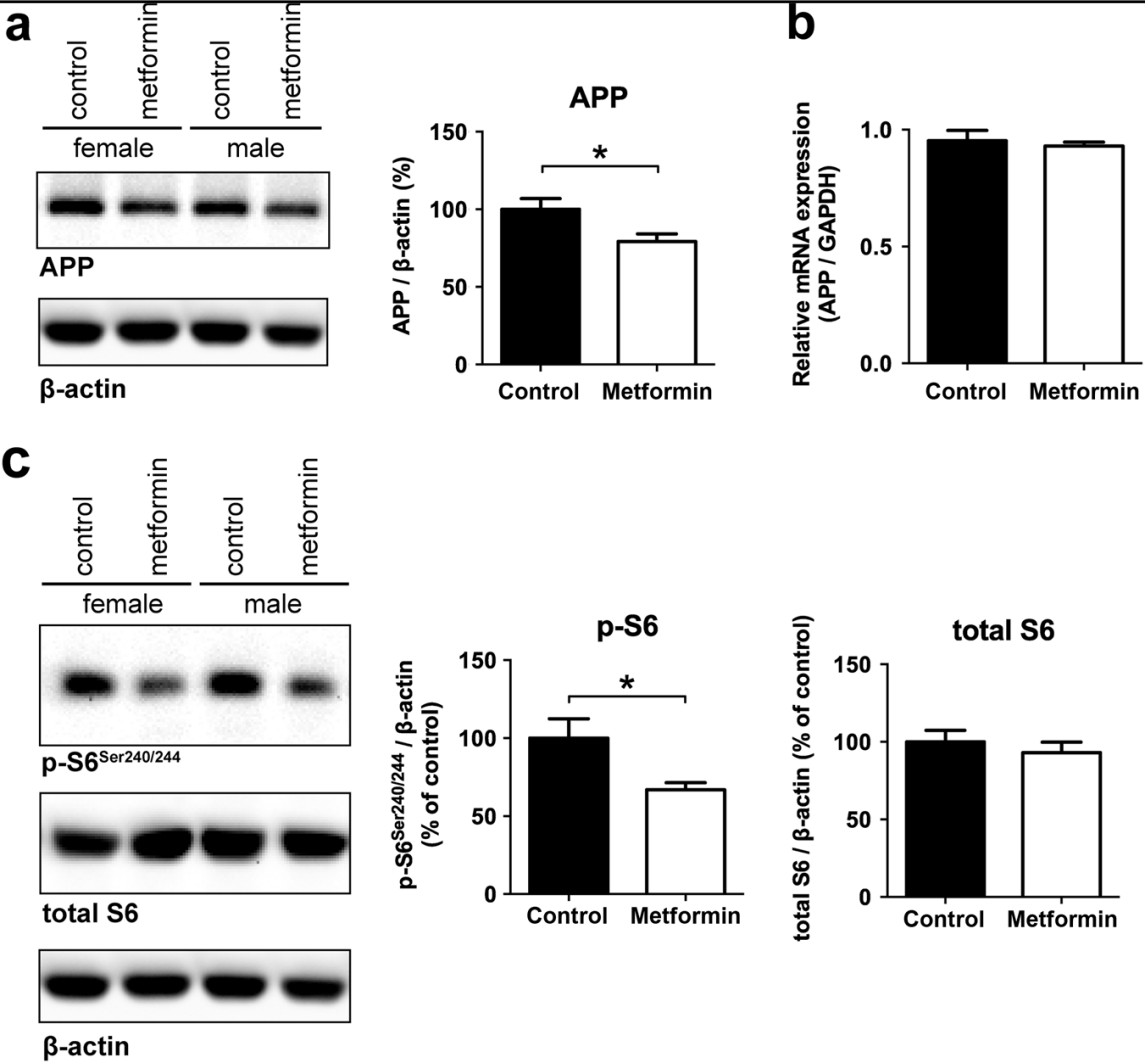
Metformin reduces APP protein level in mice. Male and female APP/PS1 mice (age 12–13 months) were treated for 8 months with 5 g/l metformin in the drinking water **a** Protein extracts from brain tissue of these animals were analyzed on western blots detecting APP and β-actin as loading control. A representative blot of *n* = 3 males and *n* = 4 females is shown. Graphs show quantification of western blots, mean values ± SEM. * = *p* < 0.05. **b** Relative APP mRNA expression was measured in brain tissues described in **a** by means of real-time PCR. Columns represent mean values ± SEM. *n* = 5. **c** Protein extracts from brain tissue of these animals was analyzed on western blots, detecting phospho-S6 (p-S6), total S6 and β-actin as loading control. A representative blot of *n* = 6 is shown. Graphs show quantification of western blots, mean values ± SEM. * = *p* < 0.05

Finally, to investigate this effect in vivo, we chronically treated transgenic APP/PS1 mice with 5 g/l metformin in the drinking water for 8 months. In line with our data from primary neurons, full-length APP protein was significantly reduced in metformin treated mice (Fig. 4a), while APP mRNA levels were not significantly changed after metformin treatment (Fig. 4b). Together, these data suggest that the APP protein level was decreased in vivo on the translational level, which can be explained by inhibition of the MID1 complex. In line with an mTOR-MID1-PP2A-dependent down-regulation of APP in these mice, the phosphorylation of S6 was significantly reduced in brain lysates of these mice, as determined by western blots (Fig. 4c).

**Fig. 5:**
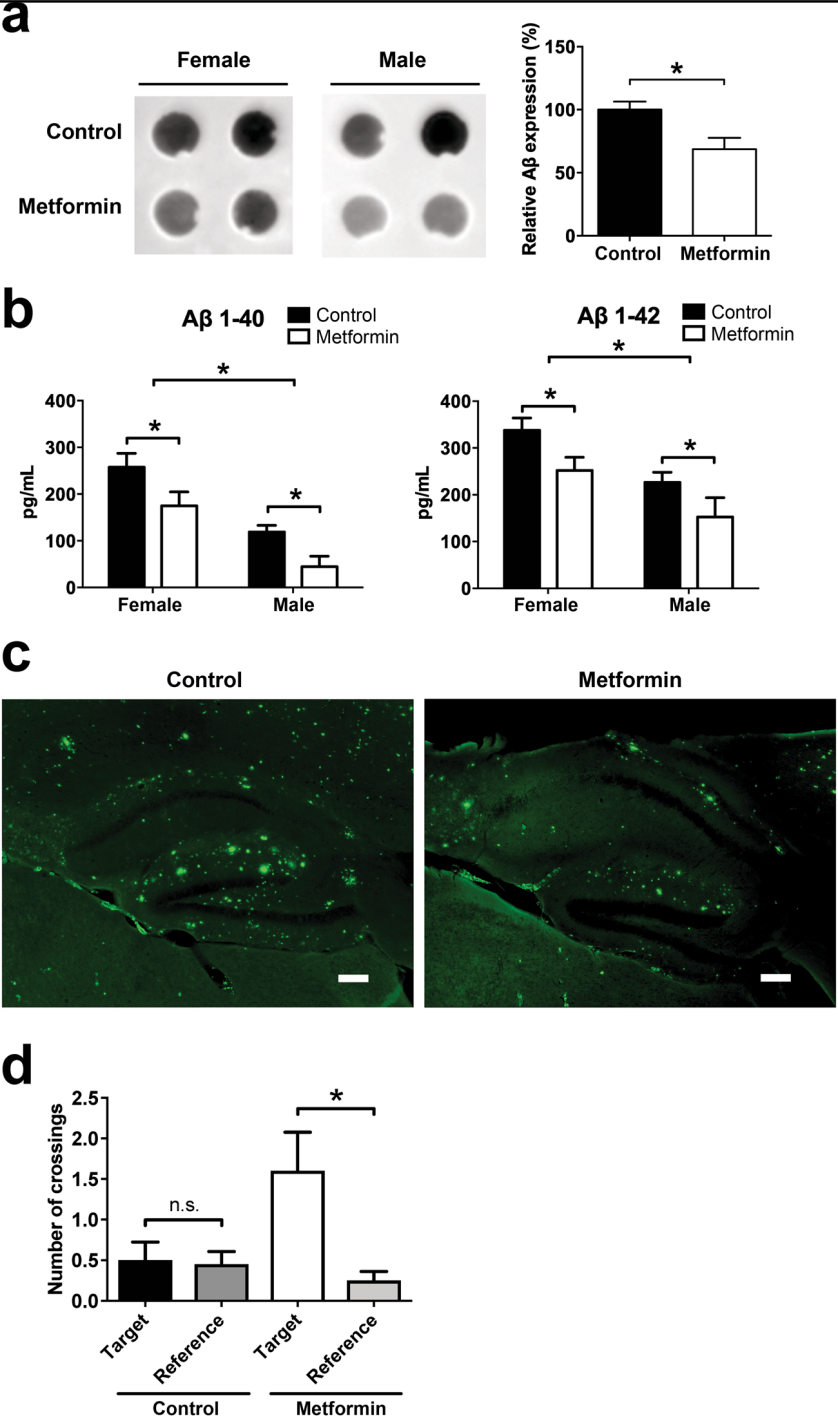
Metformin reduces Aβ plaque burden in mice. Male and female APP/PS1 mice (age 12–13 months) were treated for 8 months with 5g/l metformin in the drinking water **a** Protein extracts from brain tissue of these animals was analyzed on dot blots detecting Aβ. Representative blots of *n* = 4 females and *n* = 3 males are shown. Graphs show quantification of dot blots, mean values ± SEM, the mean value of control animals was set to 100%. *p < 0.05. **b** ELISA measurements of Aβ in brain tissues described in **a**. Columns represent mean values (pg/ml) ± SEM. *n* = 4 females, *n* = 3 males. *p < 0.05. **c** Sagittal brain sections of mice were stained with Thioflavin-S for Aβ aggregates. Scale bar = 200 µm **d** Spatial learning and memory in the Morris water maze. Mice were trained on a hidden version of the Morris water maze. After completion of training, we performed a probe trial to test how accurately the animals had learned the location of the escape platform (target). The graph shows the number of crossings of the target location vs. averaged crossings of corresponding positions in the adjacent, non-target quadrants (reference). Shown are means ± SEM. *n* = 10 mice per group. *p < 0.05

A reduction of APP should lead to a reduced Aβ plaque burden in the treated animals. To quantify this we first measured levels of Aβ on dot blots, showing that Aβ levels were significantly decreased in metformin treated animals (Fig. 5a). Second, in an ELISA measuring the levels of Aβ40 and Aβ42, a significant decrease of Aβ peptides was observed in both female and male mice (Fig. 5b). Of note, there was also a significant difference in Aβ levels between female and male mice. To investigate the Aβ plaque burden in the hippocampus, we performed Thioflavin-S stainings. In line with dot blot and ELISA experiments, thioflavin staining showed less aggregates in metformin treated animals (Fig. 5c). To test if metformin affects learning and memory, APP/PS1 mice that were treated with metformin were examined in behavioral tests. A significantly improved performance in the Morris water maze was observed in animals that were treated with metformin (Fig. 5d).

**Fig. 6:**
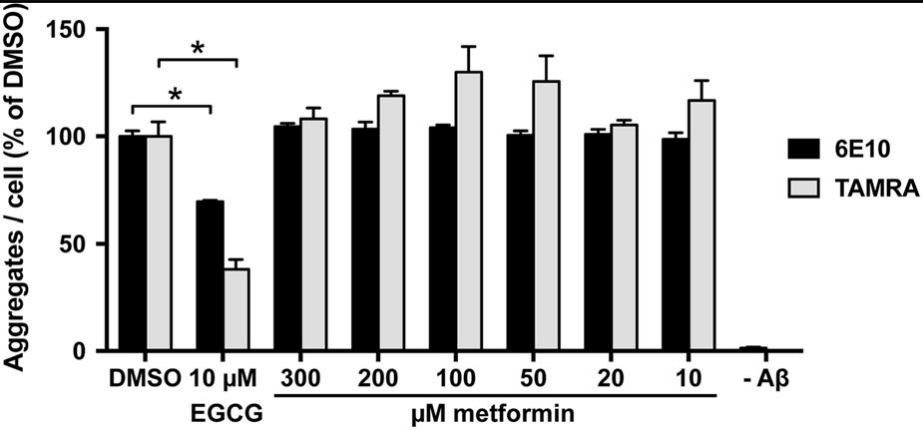
Metformin treatment does not induce degradation of Aβ Determination of cellular Aβ42 aggregate loads in SH-EP cells containing TAMRA-Aβ42 aggregates. Cells were treated with different amounts of metformin and Aβ42 aggregate load was quantified by automated fluorescence microscopy. Aggregates per cell were either quantified by the TAMRA-labeling or alternatively by immunofluorescence using the 6E10-Aβ-antibody. ECGC, a substance that was previously reported to remodel Aβ fibrils^32, 33^, was used as positive control. Graph show mean values ± SEM. *n* = 3. *p < 0.001

Besides regulating translation, mTOR is also a known regulator of autophagy. Therefore, in our in vivo set-up we cannot rule out that the observed reduction of Aβ could be at least partially due to increased clearance via autophagy. To address this, we analyzed the degradation of Aβ in SH-EP cells containing TAMRA-Aβ42 aggregates. Clearly, no increased degradation of Aβ was detectable in these cells, suggesting that metformin treatment does not induce autophageal degradation of Aβ (Fig. 6).

In summary, all these data suggest that metformin inhibits the MID1-dependent translation of APP and thereby reduces Aβ plaque burden and improves cognitive impairments in an AD mouse model.

## Discussion

In this study, we show a novel regulatory mechanism controlling the protein synthesis of APP: this mechanism involves the MID1 protein, which induces the translation of APP by regulating mTOR-eIF signaling. Disassembly of the MID1 protein complex by metformin reduces the protein production of APP. Furthermore, we show that chronic treatment of AD mice with metformin decreases the protein level of APP and its cleavage products, including Aβ. This together with our previous observation that disassembly of the MID1 protein complex by metformin also decreases tau-phosphorylation^7^, makes MID1 a particularly interesting drug target for treating AD.

Among other effects, metformin induces PP2A activity by interfering with the assembly of the MID1-PP2A-complex^7^. Although MID1 has an inhibitory effect on PP2A^8^, it positively regulates mTOR^9^. Therefore, metformin activates PP2A, while at the same time it suppresses mTOR. Decreased mTOR signalling results in activation of autophagy and decreased translation of mRNAs regulated by its downstream effectors S6K and 4E-BP1^21^. The MID1 protein complex via PP2A and mTOR stimulates translation of mRNAs that are associated with this protein complex, some of which play a role in neurodegeneration^11–13,16^.

Here we identified APP mRNA as a novel binding partner of MID1, suggesting that the MID1 complex also induces its translation. Since the biguanide metformin interferes with the assembly of the MID1 protein complex, it thereby reduces translation of the APP mRNA, leading to decreased processing by the amyloidogenic pathway. This mode of action of metformin has also been shown for other mRNAs that are associated with the MID1-complex, including the androgen receptor (AR) and BACE1 mRNA^14,15^. Furthermore, we report a so far unknown additional connection between MID1 and the mTOR-dependent translation initiation pathway: MID1 binds to RPLP0, a protein of the large ribosomal subunit. Metformin treatment interferes with the MID1-complex assembly and thus inhibits MID1-dependent translation^7,14,15^. Overall, metformin seems to decrease translation of several MID1-PP2A-mTOR dependent mRNAs. However, since chronic administration of metformin is well tolerated in type 2 diabetes patients, this does not seem to be particularly deleterious. Additionally, both induction of autophagy and reduced protein translation are especially vital for the adult nervous system, since these processes control homeostasis of Aβ and phospho-tau^19,20^. Therefore, we believe that reduced protein translation of several mRNAs regulated by MID1/PP2A/mTOR would be beneficial.

The use of metformin as a putative drug for treating AD has been discussed controversially^15,21–27^. While in some studies metformin treatment increased APP^27^ or Aβ levels^24^, other studies showed that metformin attenuated AD-like neuropathology for example by decreasing the level of the APP processing enzyme BACE1^15^, or by decreasing tau hyperphosphorylation^7,25^. This discrepancy can be explained by differences in the experimental set-up in the different studies. First, the effect of metformin seems to be dose- and time-dependent. For example, in cell culture models high doses of metformin (5–50 mM) or long incubation times result in an increased expression of APP and BACE1^24,27^, while lower doses (1–2.5 mM) decrease BACE1 protein levels as well as APP cleavage products and tau phosphorylation^7,15,23^. In wildtype mice treatment with 2 g/l metformin in the drinking water results in increased expression of BACE1 and APP as well as APP cleavage products^24,27^, while treatment with 5 g/l reduces BACE1 protein expression^15^ as well as tau-phosphorylation^7^. Also the period of treatment seems to be important. Different to studies in which increased APP and BACE1 levels were detected^24,27^, our study lasted over a treatment period of 8 months. Therefore, differences between acute and chronic pharmacological treatments could account for the observed effects. Of note, in our experiments we detected a significant difference in Aβ levels between female and male mice, with female mice having an increased Aβ burden compared to male mice. This observation is in line with previous findings, showing that female mice exhibit much higher γ-secretase activity in aged brain compared to male mice and therefore, Aβ plaque pathology in female mouse models of AD is increased compared to males^28^. Interestingly, another study in which metformin had been used in a chronic treatment demonstrated that learning and memory were improved by metformin treatment in female mice, while it had an opposite effect in male mice^26^. This shows that also the sex of the experimental animals used could account for different findings. Another important point is the age of the experimental animals in which the treatment was initiated. To our knowledge our study is the first study in which metformin treatment was initiated in aged mice displaying an already progressed state of the disease.

Taken together, our study shows that long-term treatment with metformin inhibits the MID1-dependent translation of APP and thus reduces Aβ plaque burden without any side effects for the animals. The administration of metformin for a prolonged period (8 months) started late in life and in an already progressed state of the disease. Therefore, our data represent the effects of metformin on biochemical and cognitive changes of the CNS in a progressed disease stage of AD. In addition, we could show in our previous work, that disassembly of the MID1 protein complex by metformin also decreases tau-phosphorylation^7^, making the MID1 complex a particularly interesting target for treating all AD neuropathologies.

## Materials and methods

### In vitro translation

To create an hAPP-Luciferase fusion construct, the human APP wild type splice variant 695 cDNA sequence was amplified by PCR from the pcDNA-hAPP695wt plasmid using primers APP-pGL3m-fwd and APP-pGL3m-rev (Table 2), thereby creating HindIII and NcoI restriction sites, which were used to insert the amplified sequence into the pGL3m plasmid^11^ 5′ of the firefly luciferase sequence. To enable in vitro transcription, a T7 site was inserted by PCR using primers T7-hAPP-ivts-fwd and pGL3–2258-ivts-rev (Table 2). The resulting amplificate was phenol-chloroform purified and subjected to in vitro transcription using the RiboMAX^TM^ Large scale RNA production system-T7 (Promega, Mannheim, Germany) according to the manufacturer’s instructions. In vitro transcribed RNA was phenol-chloroform purified and translated in vitro using the Flexi Rabbit Reticulocyte Lysate System (Promega) in presence or absence of inhibitors. Luciferase activity was quantified using the Firefly Luciferase Assay System (Promega) on a FLUOstar Omega 96-well plate reader (BMG Labtech, Ortenberg, Germany).

**Table 2:** Primer sequences

mAPP-RT-fwd	CAC ATC GTG ATT CCT TAC CG
mAPP-RT-rev	GTC TCA CAA ACA TCC ATC CG
mGAPDH-RT-fwd	GCA CAG TCA AGG CCG AGA AT
mGAPDH-RT-rev	GCC TTC TCC ATG GTG GTG AA
APP-pGL3m-fwd	TGC AAA AAG CTT GGC ATT CCG GTA CTG TTG GTA AAG CCA
APP-pGL3m-rev	CGT CTT CCA TGG CGC CTG GAC CGT TCT GCA TCT GCT CAA AGA ACT TGT AGG T
T7-hAPP-ivts-fwd	CGA AAT TAA TAC GAC TCA CTA TAG GGG TAA AGC CAC CAT GCT GCC CGG TTT GGC ACT GC
pGL3-2258-ivts-rev	CCG CGC CCA CCG GAA GGA GCT GAC TGG

### Immunoprecipitation

Cells have been authenticated by PCR-single-locus-technology (service by Eurofins (Ebersberg, Germany)) in December 2016. HEK293T cells were transfected with pCMV-MID1-Tag2A using PolyFect (Qiagen, Hilden, Germany) according to the manufacturer’s instructions. Untransfected cells were used as control. Cell pellets were lysed in TKM buffer (20 mM Tris pH 7.4, 100 mM KCl, 5 mM MgCl_2_, 0.5% NP40, 1 mM DTT, protease inhibitors) using a Precellys cell homogenizer. For pre-clearing, 200 µl of IgG-agarose beads were added to the lysates and incubated rotating for 30 min at 4 °C. The beads were pelleted for 5 min at 21,000 x g. Precleared lysates were then added to 200 µl anti-FLAG M1 Agarose Affinity Gel (Sigma-Aldrich/Merck, Darmstadt, Germany). After overnight rotation at 4 °C, the beads were washed 6 times and resuspended in 50 µl 1x SDS Buffer and boiled for 10 min at 95 °C. The proteins were then either identfied by mass spectrometry analysis or analyzed on a western blot. For ribosome disassembly, immunoprecipitation was performed in TKM buffer containing 40 mM EDTA. For RNase digest, the beads were washed three times after overnight incubation, anti-FLAG beads were split into two aliquots and resuspended in NEBuffer 3 (B7003S, New England Biolabs, Frankfurt, Germany). RNase If (M0243, New England Biolabs) was added to a final concentration of 500 U/ml to one immunoprecipitate and incubated for 45 min at 37 °C. Subsequently, beads were washed three times and treated as described above.

### Mass spectrometry

The eluted proteins were concentrated into one band on an SDS-PAGE gel. The band was excised and the proteins contained were processed using an automated sample preparation setup^29^. The generated peptides were purified on StageTips^30^. Samples were measured on a Q-Exactive mass spectrometer (Thermo-Fisher, Waltham, MA, USA) coupled to a Proxeon nano-LC system (Thermo-Fisher) in data-dependent acquisition mode, selecting the top 10 peaks for HCD fragmentation. A 1h gradient (solvent A: 5% acetonitrile, 0.1% formic acid; solvent B: 80% acetonitrile, 0.1% formic acid) was applied for the samples using an in-house prepared nano-LC column (0.075 mM × 150 mM, 3 μm Reprosil C18, Dr. Maisch GmbH, Ammerbuch-Entringen, Germany). A volume of 2 μl sample was injected and peptides were eluted with 3 h gradients of 5–75% solvent B at flow rates of 0.25 μl/min. MS acquisition was performed at a resolution of 70,000 in the scan range from 300 to 1700 *m*/*z*. The normalized collision energy was set to 26 eV. The mass window for precursor ion selection was set to 2.0 *m*/*z*. The recorded spectra were analyzed using the MaxQuant software package (Version 1.3.0.5)^31^ by matching the data to the Uniprot human database (downloaded on 06.05.2012) with a false discovery rate (FDR) of 1%.

### Peptide treatments

Murine primary cortical neurons were treated with 2.5 µM of a peptide that mimics the MID1-α4 binding site and thus outcompetes MID1 from binding to α4-PP2Ac. As control a mutant peptide was used. Peptides (GSK’364A and GSK’365A) containing a 29-residue sequence from α4 (AQAKVFGAGYPSLPTMTVSDWYEQHRKYG and AQAKVFGAGYPSLPTMTVSDWAEQHRKYG, respectively) with an N-terminal sequence derived from HIV-TAT protein (RKKRRQRRR) were supplied by Cambridge Research Biochemicals (Billingham, UK). They were synthesized using standard automated solid-phase peptide synthesis via the Fmoc/tBu strategy. Cleavage from the resin was performed using 95% trifluoroacetic acid. Crudes were purified by preparative high-performance liquid chromatography (HPLC), freeze dried and characterized by high-performance liquid chromatography (HPLC) and matrix-assisted laser desorption ionization time-of-flight (MALDI-TOF) mass spectrometry.

### In vivo treatments mice

Male and female APP/PS1 (B6C3-Tg(APPswe,PSEN1dE9)85Dbo/Mmjax) mice (age 12–13 months) were treated for 8 months with 5 g/l metformin in the drinking water with daily change of water and addition of fresh metformin. Water intake and body weight of the animals were monitored. After 8 months of treatment, animals were sacrificed and brains were snap-frozen in liquid nitrogen and broken up using a mortar. All procedures were in compliance with German Animal Protection Law and were approved by the competent authorities (Landesamt für Naturschutz und Verbraucherschutz Nordrhein-Westphalen; AZ 87–51.04.2011.A049/01).

### Morris water maze

We assessed spatial learning and memory in the Morris water maze in APP/PS1 mice treated with metformin or vehicle control. The water pool (Med Associates) had a diameter of 1.2 m and was filled with opaque water (temperature: 24 °C). Mice received 6 daily training trials for 3 consecutive days on a hidden version of the Morris water maze (i.e., the maze contained an escape platform hidden underneath the water surface in a constant location of the pool). To evaluate the accuracy with which the animals had learned the position of the escape platform, we performed a probe trial (during which the platform was removed from the pool) once training was completed. Behavior of the animals was recorded using an automated tracking system (Ethovision XT, Noldus). We determined the number of crossings of the exact target location (i.e., where the platform was located during training) and compared it to the average crossings of analogous positions in the adjacent, non-target quadrants (reference).

### Thioflavin-S staining

Sagittal brain sections were incubated in 1x TBS buffer containing 10% Triton X-100 and transferred onto glass slides. The slides were dried overnight at room temperature. Slides were washed 3 times for 3 min in distilled water, and incubated for 3 min in 0.1% Thioflavin-S staining solution (dissolved in 10% ethanol diluted in distilled water) in the dark. Sections were washed 3 times in distilled water and incubated for 20 min in 1% acetic acid in the dark. Slides were washed with tap water, cover-slipped with mounting medium (Thermo-Fisher), and stored in the dark at 4 °C.

### Western blot

Brain samples were homogenized either in RIPA buffer (20 mM Tris-HCl (pH 7.5), 150 mM NaCl, 1 mM EDTA, 1 mM EGTA, 1% NP-40, 1% sodium deoxycholate, 2.5 mM sodium pyrophosphate, 1 mM β-glycerophosphate, 1 mM Na_3_VO_4_, 1 μg/ml leupeptin) or in SDS PAGE buffer B (40 mM Tris-HCl pH 6.8, 4% Glycerol, 2% SDS, 0.01% bromophenolblue, 2 mM 2-mercaptoethanol), sonicated and boiled for 5 min at 95 °C. Proteins were analyzed on 10 or 12% SDS gels and blotted onto PVDF membranes (Roche, Mannheim, Germany). Blots were blocked in milk and incubated with the antibodies listed below.

Bands were densitometrically quantified using AIDA software v4.27 (Raytest, Straubenhardt, Germany).

### Statistical analysis

Statistical analyses were performed using two-way ANOVA, as well as Student’s t-test or Mann-Whitney test (two-tailed) for two-group comparisons, as appropriate.

### Dot blot

Brain samples were homogenized in RIPA buffer. 50 µg total protein per well were loaded and proteins were transferred to a PVDF membrane using a HYBRI-DOT manifold. The aggregates on the membrane were detected by incubation with anti-beta-amyloid 6E10 antibodies (BioLegend, San Diego, CA, USA).

### ELISA

ELISA assays to measure Aβ were performed using the Aβ40 / Aβ42 ELISA Kits (Life Technologies) according to the manufacturer’s protocol.

### Antibodies

The following antibodies were purchased from Cell Signaling (Leiden, Netherlands): S6 (#2317), pS6 (#4858), actin (#4967), eIF3A (#3411), eIF4G (#2498), RPL5 (#51345) and GAPDH (#2118). FLAG-HRP (A8592) antibody was purchased from Sigma; RPLP0 (ab192866), RPS3 (#128995), and APP (ab2071) from Abcam (Cambridge, UK), and anti-beta-amyloid 6E10 from BioLegend (803001).

### Real-time PCR

Total RNA was isolated using the RNeasy Plus Mini Kit (Qiagen). cDNA was synthesized using the TaqMan reverse transcription reagents kit (Applied Biosystems, Waltham, MA, USA) and real-time PCR was carried out using the SYBRGreen PCR master mix (Applied Biosystems). Primers used are listed in Table 2.

### RNA immunoprecipitation

Murine primary cortical neurons were transfected with FLAG-tagged MID1 using Lipofectamine 2000 (Invitrogen Waltham, MA, USA). 48 h after transfection, cells were treated with or without 2.5 mM metformin and incubated another 24 h. After UV-crosslinking (200 mJ/cm^2^) cells were lysed in TKM buffer (20 mM Tris pH 7.4, 100 mM KCl, 5 mM MgCl_2_, Complete protease inhibitor cocktail (Roche), RNAse inhibitor, 0.2% NP40) and MID1 protein complexes were purified by immunoprecipitation using anti-FLAG M1 Agarose Affinity Gel (Sigma-Aldrich) or IgG-agarose (Sigma-Aldrich) as a negative control. Protein-bound mRNA was isolated after DNAse and proteinase K digestion by phenol-chloroform purification and analyzed by RT-PCR. Primers used are listed in Table 2.

### Automated fluorescence microscopy and determination of cellular Aβ42 aggregate loads

For fluorescent labeling of Aβ aggregates, 20 µM Aβ42 peptide stock solutions diluted in low salt buffer (10 mM NaCl, 1.9 KH_2_PO_4_, 8.1 mM K_2_HPO_4_, pH 7.4) were mixed with 5% Aβ42 peptides which have been N-terminally labeled with the fluorophore 5-Carboxytetramethylrhodamine (TAMRA) in solid-state peptide synthesis by AnsSpec, Fremont, USA. Then, mixed Aβ peptide solutions were aggregated at 37 °C for 18 h under 300 rpm constant agitation followed by sonication with a Sonic Dismembrator Model 120 from Fisher Scientific GmbH (Schwerte, Germany) at low intensity for 6 rounds of 10 s. SH-EP cells (DSMZ, Braunschweig, Germany) were cultured in DMEM (Gibco by Thermo-Fisher GmbH, Dreeich, Germany) containing 10% fetal bovine serum (FBS), 5% Glucose, 100 units/ml penicillin and streptomycin, respectively. Incubation was carried out at 37 °C with 5% (v/v) CO_2_. For Aβ42 aggregate internalization, cells were treated with 600 nM or 1 µM TAMRA-Aβ42 for 18 h. To ensure removal of free and surface-bound aggregates, Aβ containing medium was aspirated, cells were washed with phosphate-buffered saline (PBS), trypsinized and collected in fresh medium. Then, cells were seeded into 96-well cell culture plates and treated with different amounts of metformin for 6 h. Cells were fixed in 2% paraformaldehyde for 20 min at room temperature, followed by Nuclei staining with Hoechst (1:2500 Hoechst 33342, Sigma-Aldrich Chemie Gmbh Munich, Germany) and then washed twice with PBS, before fluorescent microscopy was performed in a Cellomics ArrayScan Hight-Content System (Thermo-Fisher) using an objective with 20-fold magnification. After image acquisition, automated data analysis was performed using ArrayScan VTI (700 Series, Thermo-Fisher). For quantification, individual cells were detected via Hoechst fluorescent signals (XT53, filter and dichroic-emitter pair) and total TAMRA fluorescent areas per cell (XT32, filter and dichroic-emitter pair) were measured and calculated from technical triplicates. Alternatively, Aβ was stained by immunofluorescence using the 6E10 antibody (BioLegend).
